# Supplementary material for: EndoBind detects endogenous protein-protein interactions in real time
Source: Commun Biol. 2021 Sep 15;4:1085. doi: 10.1038/s42003-021-02600-5 (PMC8443649; doi:10.1038/s42003-021-02600-5)
Supplement: Supplementary file 2 — Supplementary Information [file 42003_2021_2600_MOESM2_ESM.pdf]

EndoBind detects endogenous protein-protein interactions in real time

Anke Bill*[1], Sheryll Espinola[2], Daniel Guthy[3], Jacob R. Haling[2,4], Mylene Lanter[3], Min Lu[2], Anthony Marelli[2], Angelica Mendiola[2], Loren Miraglia[2], Brandon Taylor[2], Leonardo Vargas[2], Tony P. Orth[2], Fred King*[2]

** corresponding authors (anke.bill@novartis.com, fred.king@novartis.com)*

[1]*Novartis Institute for Biochemical Research, Oncology, Cambridge, MA, USA*

[2]*Genomics Institute of the Novartis Research Foundation, Assay Development and High Throughput Screening, San Diego. CA, USA*

[3]*Novartis Institute for Biochemical Research, Oncology, Basel, Switzerland*

[4]*Current: Mirati Therapeutics, Inc., Research, San Diego, CA, USA*

Supplementary Figure 1

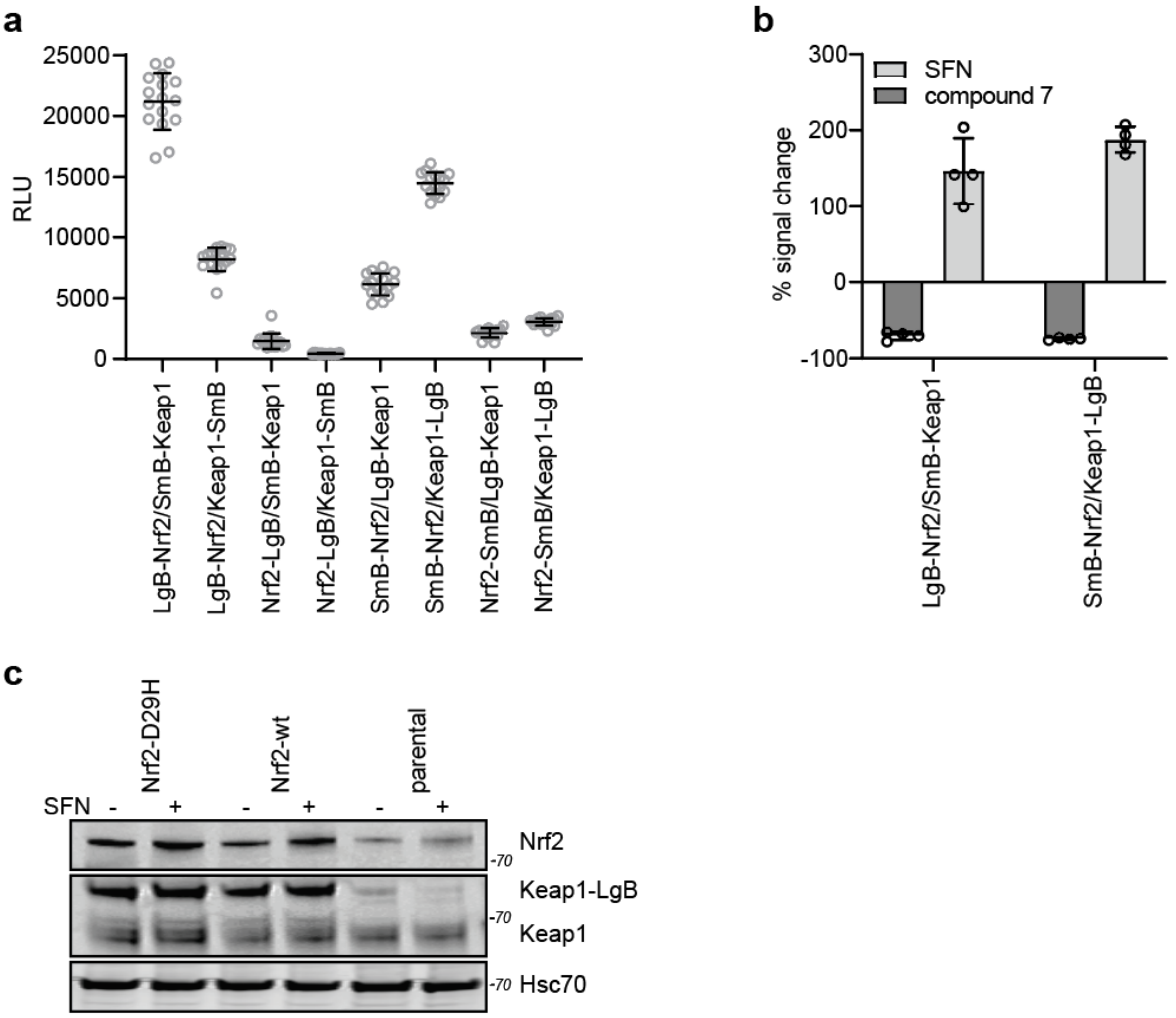

Supplementary Figure 1. **a**. Luminescence signal measured for HEK293T cells stably expressing the indicated constructs (mean ± s.d., n=16 wells each, representative experiment) as detected after addition of NanoGlo. **b**. Relative change in luminescence signal after 4 h treatment with 10 µM Sulforaphane (SFN) or 1 µM compound 7 in the indicated cell lines (mean ± s.d., n=3 wells each of a representative experiment) as detected after addition of NanoGlo. Data was normalized to the treatment control (DMSO). **c.** Protein levels of indicated proteins in HEK293T- SmB-Nrf2/Keap1-LgB or SmB-Nrf2-D29H/Keap1-LgB cells treated for 4 h with DMSO or 10 µM SFN detected by western blotting. Representative image is shown.

Supplementary Figure 2

a

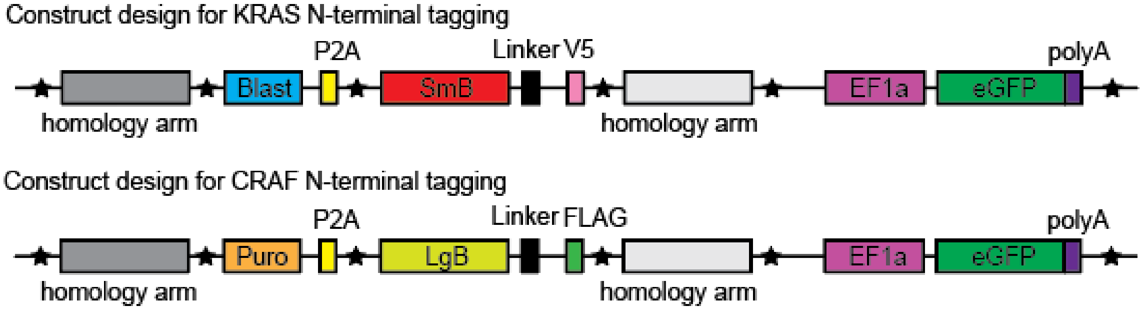

b

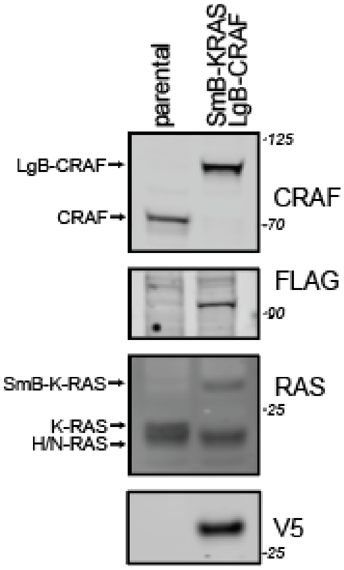

c

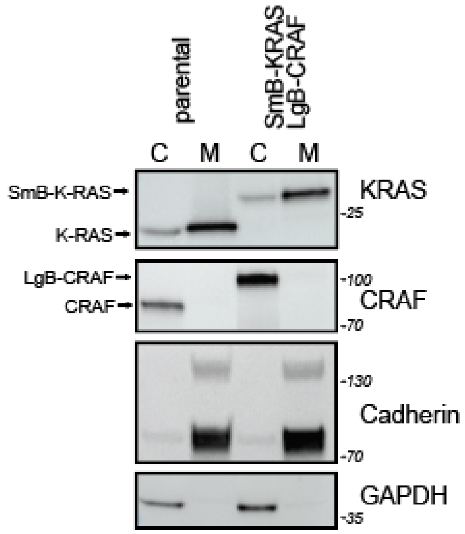

d

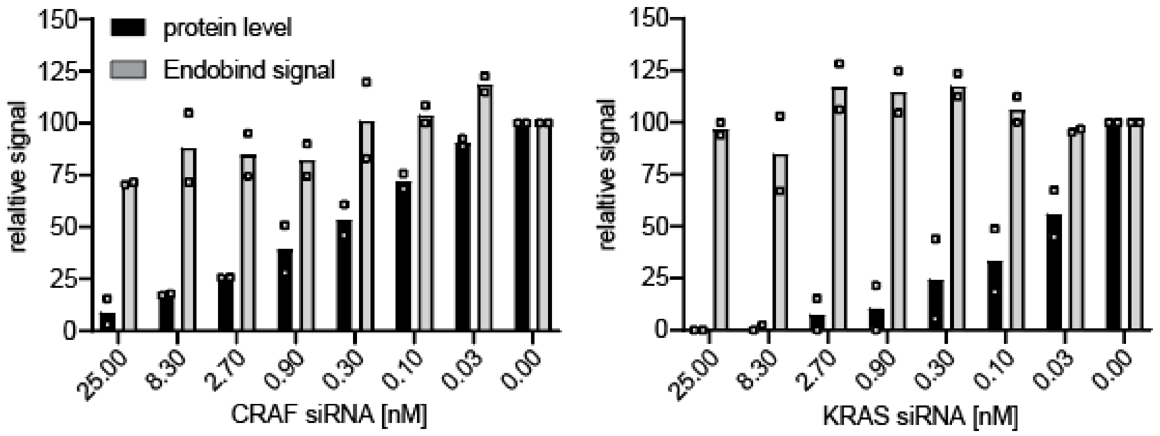

Supplementary Figure 2. **a**. Schematic design of the donor vectors used for integration of the SmB or LgB at the N-terminus of KRAS and CRAF, respectively. The vectors contain a selection cassette followed by a P2A site, the SmB or LgB sequence, a short linker and a V5- or FLAG-tag, flanked by 800bps homology arms. An EF1alpha-promotor followed by an eGFP-sequence enabled us to select for cells with unspecific integration of the vector sequence (=GFP positive cells). **b.** Protein levels in PATU8988T-SmBKRAS-LgBCRAF and parental PATU8988T cells as detected by western blotting. Note: The pan-RAS antibody detects all RAS forms. The lower band corresponds to H- and NRAS, while the upper band corresponds to KRAS[1]. Note the upshift of the KRAS-band after addition of the tag. **c.** Protein levels in PATU8988T-SmBKRAS-LgBCRAF and parental Patu8988T cells as detected by western blotting after fractionation. Cadherin was used as membrane (M) marker while GAPDH was used as a cytosolic (C) marker. **d.** EndoBind signal after knockdown of KRAS or CRAF in PATU8988T-SmBKRAS-LgBCRAF cells without GDC0879 treatment (mean ± s.d., n=3 for siKRAS and mean of n=2 for siCRAF). Signals were normalized to cells transfected with a non-targeting siRNA. Protein levels after transfection of the indicated amount of siRNA were quantified using the Jess system (mean, n=2).

Supplementary References

1. Waters, A.M. et al. Evaluation of the selectivity and sensitivity of isoform- and mutation-specific RAS antibodies. *Sci Signal* **10** (2017).
