# Supplementary material for: EndoBind detects endogenous protein-protein interactions in real time
Source: Commun Biol. 2021 Sep 15;4:1085. doi: 10.1038/s42003-021-02600-5 (PMC8443649; doi:10.1038/s42003-021-02600-5)
Supplement: Supplementary file 3 — Description of Additional Supplementary Files [file 42003_2021_2600_MOESM3_ESM.pdf]

Description of Additional Supplementary Files

File name: Supplementary Data 1.

Description: Source data for all figures.
